# Multi-locus variable-number tandem repeat analysis for outbreak studies of *Salmonella enterica *serotype Enteritidis

**DOI:** 10.1186/1471-2180-8-84

**Published:** 2008-05-30

**Authors:** Burkhard Malorny, Ernst Junker, Reiner Helmuth

**Affiliations:** 1 Federal Institute for Risk Assessment, National Salmonella Reference LaboratoryDiedersdorfer Weg 1, D-12277 Berlin, Germany

## Abstract

**Background:**

*Salmonella enterica *subsp. *enterica *serotype Enteritidis is known as an important and pathogenic clonal group which continues to cause worldwide sporadic cases and outbreaks in humans. Here a new multiple-locus variable-number tandem repeat analysis (MLVA) method is reported for highly-discriminative subtyping of *Salmonella *Enteritidis. Emphasis was given on the most predominant phage types PT4 and PT8. The method comprises multiplex PCR specifically amplifying repeated sequences from nine different loci followed by an automatic fragment size analysis using a multicolor capillary electrophoresis instrument. A total of 240 human, animal, food and environmental isolates of *S*. Enteritidis including 23 definite phage types were used for development and validation. Furthermore, the MLVA types were compared to the phage types of several isolates from two recent outbreaks to determine the concordance between both methods and to estimate their in vivo stability. The in vitro stability of the two MLVA types specifically for PT4 and PT8 strains were determined by multiple freeze-thaw cycles.

**Results:**

Seventy-nine different MLVA types were identified in 240 *S*. Enteritidis strains. The Simpson's diversity index for the MLVA method was 0.919 and Nei diversity values for the nine VNTR loci ranged from 0.07 to 0.65. Twenty-four MLVA types could be assigned to 62 PT4 strains and 21 types to 81 PT8 strains. All outbreak isolates had an indistinguishable outbreak specific MLVA type. The in vitro stability experiments showed no changes of the MLVA type compared to the original isolate.

**Conclusion:**

This MLVA method is useful to discriminate *S*. Enteritidis strains even within a single phage type. It is easy in use, fast, and cheap compared to other high-resolution molecular methods and therefore an important tool for surveillance and outbreak studies for *S*. Enteritidis.

## Background

*Salmonella enterica *subspecies *enterica *serovar Enteritidis (*S*. Enteritidis) is the world-leading cause of salmonellosis and is often implicated in over 60% of cases of human salmonellosis in Europe [1]. In the United States it remains the second most common serotype of salmonellae [2]. The current worldwide epidemic of *S*. Enteritidis started in the middle of the 1980s [3]. The reservoir for *S*. Enteritidis is mainly poultry often carrying asymptomatic infections, which pass the human pathogen along the food production chain. Especially undercooked or raw eggs and frozen poultry meat represent a high risk for humans.

*S*. Enteritidis is one of the most clonal *Salmonella *serotype [4,5]. Consequently, highly discriminative typing methods are needed for surveillance and outbreak studies. Phage typing is the traditional phenotypic method for subtyping salmonellae but has limited discriminative power and requires standardized phage collections [6]. Molecular-based typing methods like plasmid profiling, RAPD and pulsed-field gel electrophoresis (PFGE) have been applied with limited discriminatory power as well [7]. Ribotyping using the restriction enzymes *Pst*I and *Sph*I is currently the most useful method for discrimination [8]. However, this method is extremely labor-intensive and difficult to standardize. Advances in the Polymerase Chain Reaction (PCR) technology has resulted in the development of the multiple-locus variable-number of tandem repeat analysis (MLVA). It is a new approach to subtype bacteria which involve amplification and fragment size analysis of polymorphic regions of DNA containing variable numbers of tandem repeat sequences. This method has been found to be very useful in discriminating between isolates that are highly clonal including various pathogenic species [9]. For *Salmonella*, MLVA systems for the species *enterica *[10], for the serotype Typhimurium [11] and the serotype Enteritidis [12] have been described. Several outbreak studies for Typhimurium have shown the usefulness of this method to identify the source of infection [13,14] and were superior to PFGE in the discrimination power [15].

Here we report a new multicolor MLVA method for *S*. Enteritidis which is highly discriminative, fast and easy to use. One multiplex PCR per strain using fluorescently labeled primers is performed and the individual PCR fragment sizes are analysed by a multicolor capillary electrophoresis system. For each locus fragment sizes are assigned to allele numbers as the basis for strain identification. Emphasis was given on phage types 4 (PT4) and 8 (PT8) which are the most prevalent phage types in Europe [16] causing sporadic and outbreak cases. Investigations of additional 52 isolates sampled from two recent outbreaks showed the correlation with phage typing and other molecular methods.

## Results

### Diversity of *S. Enteritidis *isolates analysed by MLVA

Two-hundred and forty epidemiologically independent *S*. Enteritidis isolates belonging to 23 different phage types were arbitrarily selected and analysed by MLVA. All nine VNTR loci selected (Table 1) were specifically amplified in one multiplex PCR using fluorescently labeled primers (Table 2). Afterwards, the products were separated according to their size using the ABI310 multicolor capillary electrophoresis Genetic Analyser. Altogether 79 different allele combinations (MLVA types) have been found among the 240 *S*. Enteritidis strains tested. The Simpson's diversity index for MLVA was 0.919 and for phage typing 0.813. The VNTR loci SENTR4, SENTR5, SENTR6 and SE-7 showed similar Nei's diversity values of 0.6 (Table 3). Of these in SENTR 5 (10 alleles) and in SENR 6 (11 alleles) the highest number of different tandem repeat alleles (11 alleles) were detected. The lowest Nei's diversity values were observed for the loci SENTR 1, SENTR 2 and SENTR 3 (each 3 alleles) (Table 3). However, despite the low diversity values these loci were valuable to discriminate strains of certain phage types. Particularly, VNTR variants in SENTR 2 occurred in 7 of the 81 tested PT8 isolates and variants in SENTR 3 were able to discriminate PT4, PT8 and PT26 isolates. Null variants (no fragment) have been observed for the locus SE-3 in PT13, PT7a and PT9b, for the locus SENTR 4 in PT26, and for the locus SENTR 3 in PT4, PT8 and PT9b strains.

**Table 1:** Characteristics of VNTR loci selected for MLVA

Locus name (locus name in Boxrud et al. [12])	Repeat size (bp)	No. of repeats in strains		Region
			
		NCTC 13349^a^	07–2642^b^	
SENTR1 (SE-10)	45	7.8	7.8	*tolA*
SENTR2^c^	39	9.3	9.3	*ftsK*
SENTR3	93	4.4	4.4	STM1457 homologue
SENTR4 (SE-1)	7	4.9	4.9	Putative bacteriophage tail protein, STY631a homologue
SENTR5 (SE-5)	6	10.0	10.0	*yohM*
SENTR6 (SE-2)	7	5.1	5.1	non-coding
SENTR7 (SE-9)	9	3.1	3.1	*ushA*
SE-3^d^	12	3.0	3.0	non-coding
SE-7^d,e^	61^3^	8.5	8.5	*ygbF*

^a ^*S*. Enteritidis NCTC 13349 [25].

^b ^number of repeats confirmed by DNA sequencing, DNA sequences identical to NCTC 13349.

^c ^SENTR2 corresponds to Locus STTR7 in Lindstedt et al. [22].

^d ^adapted from Boxrud et al. [12].

^e ^repeat units consist of a 29 bp highly conserved 5'-sequence and a 32 bp variable 3'-sequence.

**Table 2:** Primer sequences and PCR product size used for *S*. Enteritidis typing

Locus	Primer	Dye-Sequence	DNA sequence length in bp^a ^(size ABI 310^b^)
SENTR1	SENTR1-F	**HEX**-GCAACAGCAGCAGCAACAG	440 (415)
	SENTR1-R	CCGAGCTGAGATCGCCAAG	
SENTR2	SENTR2-F	**FAM**-CACTGGACGATCTGGATTTCTC	519 (510)
	SENTR2-R	GTCGCCGTTACGCATCAAC	
SENTR3	SENTR3-F	**TET**-CTAAACAAGCCGCTCATCCG	494 (480)
	SENTR3-R	ACAACCTGCTGCTGTGCTG	
SENTR4	SENTR4-F	**HEX**-GACCAACACTCTATGAACCAATG	120 (120)
	SENTR4-R	ACCAGGCAACTATTCGCTATC	
SENTR5	SENTR5-F	**FAM**-CACCGCACAATCAGTGGAAC	271 (270)
	SENTR5-R	GCGTTGAATATCGGCAGCATG	
SENTR6	SENTR6-F	**TET**-ATGGACGGAGGCGATAGAC	180 (177)
	SENTR6-R	AGCTTCACAATTTGCGTATTCG	
SENTR7	SENTR7-F	**FAM**-ACGATCACCACGGTCACTTC	135 (133)
	SENTR7-R	CGGATAACAACAGGACGCTTC	
SE-3	SE-3F	**HEX-**CAACAAAACAACAGCAGCAT	308 (307)
	SE-3R	GGGAAACGGTAATCAGAAAGT	
SE-7	SE-7F	**HEX-**GATAATGCTGCCGTTGGTAA	717 (691)
	SE-7R	ACTGCGTTTGGTTTCTTTTCT	

^a ^based on *S*. Enteritidis NCTC 13349 [25].

^b ^size analysed using the capillary sequence detection system ABI 310 (Applied Biosystems)

**Table 3:** Variability of selected VNTRs in 240 *S*. Enteritidis

Locus	Smallest PCR product size (bp) ^a^	Largest PCR product (bp) ^a^	No. of alleles	Nei's diversity index
SENTR1	332	418	3	0.07
SENTR2	276	511	3	0.07
SENTR3	388	574	3	0.07
SENTR4	113	133	7	0.63
SENTR5	239	326	10	0.65
SENTR6	148	232	11	0.64
SENTR7	116	134	3	0.51
SE-3	307	320	3	0.49
SE-7	635	750	6	0.63

^a ^size determined on a capillary ABI 310 Genetic Analyser

**Figure 1:**
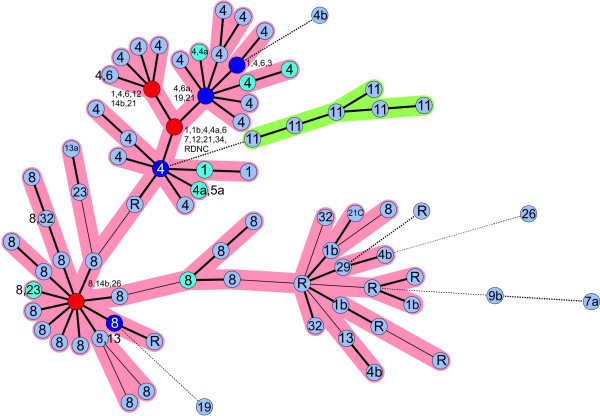
**Minimum spanning tree of 240 *S*. Enteritidis strains based on MLVA**. The categorical coefficient and the highest number of single-locus-changes have been used for the construction. Each circle represents one MLVA type. The numbers in the circles indicate the corresponding phage type(s) of the strain(s). Bold short lines link MLVA types differing by one allele. Thin lines link MLVA types with two different alleles and bold dotted lines connect types different by three alleles and thin dotted lines differ in four alleles. The different colors of the circles indicate the number of strains with a particular MLVA type: light blue circles 1 to 2 strains, turquoise circles 3 to 5 strains, purple circles 6 to 10 strains, red circles 11 to 50 strains. The green cluster represents isolates of the phage type 11 having a maximum distance of changes for three alleles.

Twenty-four different MLVA types were assigned to the 62 PT4 strains analysed and 21 different types were identified for 81 PT8 strains analysed. The most frequent type in PT4 strains was represented by allele numbers 3-4-2-4-1-3-1-2-5 (21 strains) and in PT8 strains the type 2-4-2-6-1-2-2-2-6 (45 strains) predominated. PT8 strains with the most frequent MLVA type were often isolated from eggs or egg products in northern parts of Germany in 2006 and 2007. This indicates probably the spread of a certain clone within egg-laying hens in this region. Generally, strains with the same phage type cluster together with similar MLVA types with some exceptions. PT4 and PT8 strains were separated by major branches (Fig. 1). Twelve out of 16 PT21 strains tested had the same MLVA type 3-4-2-4-1-3-1-2-5 as PT4 strains. PT6 strains have often the same MLVA type as PT4 strains. The seven PT1 strains tested were assigned to six different types but all types were also observed in PT4 strains.

### Outbreak investigations

Strains isolated from two different outbreaks were investigated using the MLVA protocol to show the applicability (in vivo stability) of the MLVA method compared to phage typing. The first outbreak occurred in summer 2006 during a sporting event. After consumption of pastry (éclairs) 46 participants developed gastrointestinal symptoms. *Salmonella *isolates have been obtained from stool samples of the patients, from utensils and pastries of the manufacturing pastry shop and the confectioner, who turned out to shed salmonellae [17]. Altogether 14 isolates were analysed by MLVA, phage typing, ribotyping and PFGE. All isolates were indistinguishable by ribotyping, PFGE and MLVA. For phage typing all but one isolate showed the same type PT4. The exceptional strain was assigned to RDNC (React Did Not Confirm) indicating a phenotypic changes of the strain. The MLVA type was assigned to 3-4-2-4-1-3-1-2-5, most frequently found in sporadic PT 4 strains tested.

The second outbreak occurred between week 4 and 24 in 2007 [18]. Twenty-eight patients acquired salmonellosis caused by *S*. Enteritidis in a German hospital. At the same time children of a kindergarten were also affected. The kindergarten received its lunch from the same hospital kitchens as the patients in the hospital. It was likely that both outbreaks were caused by contaminated foods prepared in the hospital's kitchen. Isolates were obtained from kitchen personnel shedding *S*. Enteritidis, various foods (potato soup, red fruit jelly, cheese and vanilla sauce), sick children from the kindergarten, and sick patients from the hospital. Thirty-eight isolates were typed by MLVA and phage typing. All isolates were indistinguishable in their phage type PT8 and MLVA type 2-4-2-6-1-2-2-2-6, the most frequent type found in PT8. A common source of infection could not be identified indicating that probably kitchen staff members shedding salmonellae had contaminated foods during preparation and consequently causing the outbreak. Alternatively, since the MLVA type is frequently associated with isolates from egg products it might be speculate that any contaminated eggs or egg powder were used for food preparation, also affecting staff members in the kitchen. However, suspicious foods for further analysis were not available.

### Stability of VNTR loci

The in vitro stability of the VNTR loci was tested by freeze-thaw subculturing cycles of single colonies of strains 07–2642 (PT4) and 07–3048 (PT8). One single colony of each strain was frozen in glycerol at -80°C. Ten cycles of freezing and thawing were performed (one cycle per day). Afterwards, a subculture was grown on Luria-Bertani agar and five single colonies were picked and each frozen again in glycerol at -80°C. After repeating 10 cycles of freezing and thawing the five subcultures were grown on Luria-Bertani agar again and ten single colonies were picked from each subculture. All 50 samples of one strain were determined in the MLVA. The results showed indistinguishable MLVA types as the original strain.

## Discussion

We report the first MLVA multicolor capillary electrophoresis method for the high-resolution discrimination of *S*. Enteritidis isolates. In Europe over 60% of human salmonellosis cases are caused by *S*. Enteritidis and the serotype is considered as highly clonal group. A multiplex PCR has been developed to amplify nine VNTR loci of the *S*. Enteritidis genome simultaneously. The primers were labeled with the dyes 6-FAM, HEX or TET for the accurate assignment of individual PCR products to a specific VNTR locus after multicolor capillary electrophoresis. The Geneflo-625 marker (TAMRA labeled) modified by an additional 868 bp-size fragment has been used as internal size standard. The additional fragment is necessary because PCR products of the SE-7 locus might reach sizes up to 750 bp. Alternatively, the commercial size marker MapMarker 1000 (BioVentrures, Inc.) could be used which consists of 23 discrete DNA fragments ranging in size from 50 to 1000 base pairs. Preliminary results showed no significant difference of PCR product sizes between both markers.

In most cases, isolates with the same phage type grouped together with some exceptions (Fig. 1). It has been observed that strains of different phage types can exhibit the same MLVA type. This has been observed, for example for strains with the phage type 1, 1b, 3, 12, 13, 21, 5a, 6, and 34 (Fig. 1). Therefore, the method is not necessarily suitable to predict the phage types. This has been also shown for the well-evaluated *S*. Typhimurium MLVA method according to Lindstedt et al. [11]. Consequently, the combination of traditional phage typing and the new MLVA typing can lead to an even higher typing resolution for *S*. Enteritidis.

To evaluate the discriminatory power of the MLVA method the focus was given on the phage types PT4 and PT8. Both types play a predominant role in the epidemiology of *S*. Enteritidis [16,19]. The MLVA method showed an excellent discrimination within these phage types similar to or higher than two restriction enzyme ribotyping as previously described [7,8]. The major advance of this MLVA method compared to ribotyping is its easy use, lower cost and rapidity making it valuable for outbreak studies. In addition, the exchange of data for comparative studies between laboratories is very easy as demonstrated for *S*. Typhimurium MLVA [20].

Boxrud et al. [12] have also recently published a MLVA method for *S*. Enteritidis using ten VNTR loci amplified in individual PCR reactions. This MLVA was recently optimized using a multiplex PCR for seven VNTR loci followed by a single color electrophoresis using a Beckman CEQ 8000 automated DNA sequencer [21]. However, only 34 *S*. Enteritidis strains were tested representing a maximum of variety. Due to the use of a single color electrophoresis system it is questionable if PCR products variable in sizes can be reliably assigned to the correct VNTR locus. Several VNTR loci used by Boxrud et al. [12] were independently selected by the MLVA method presented here. However, the loci SENTR2 and SENTR3 were not previously described by Boxrud et al. [12]. The locus SENTR2 was previously described as STTR7 by Lindstedt et al. [22]. Despite the low diversity index determined for these loci in regard to all 240 *S*. Enteritidis strains tested, they were useful for achievement a better diversity within PT4 and PT8 isolates.

The analysis of *Salmonella *isolates belonging to two outbreaks have shown that during the course of each outbreak a stable MLVA type was obtained. The results were in congruence with other molecular typing methods (ribotyping, PFGE) and phage typing. In addition, the determination of the in vitro stability by doing multiple freezing thawing cycles of glycerol stock cultures revealed no changes in the MLVA type. Hopkins et al. [23] observed for *S*. Typhimurium MLVA sporadically single-locus variants in strains isolated from different outbreaks. This might indicate that small changes in loci are rather likely in *S*. Typhimurium than in *S*. Enteritidis supported by the knowledge that the serotype *S*. Enteritidis is less diverse in genotypic properties than the serotype *S*. Typhimurium. Hopkins et al. [23] proposed for *S*. Typhimurium to define the cut-off to allow the classification of strains as part of an outbreak as a difference of two repeats at one or fewer loci. This rule might be adapted for *S*. Enteritidis to only one repeat in one locus because of its clonality. However, the analysis of more outbreaks is needed to determine the hypothesis.

## Conclusion

In conclusion, the MLVA method for high-resolution of *S*. Enteritidis isolates presented here offers a simple, robust and cheap tool for surveillance, outbreak studies and short-term tracing of bacteria in the food chain. Data can easily be shared between laboratories by a standardized allele string or MLVA type number which is an important advantage for outbreak studies occurring in several countries. The in vivo and in vitro stability of the VNTR loci indicate less frequent changes in the number of tandem repeats than shown for *S*. Typhimurium. This might reflect the rather clonal genome structure of this common serotype.

## Methods

### *Salmonella *strains

For the development of a MLVA method 240 *Salmonella *Enteritidis strains isolated between 1992 and 2007 were selected from the strain collection of the National Salmonella Reference Laboratory, Germany. The strains originated from animals, foods, environmental and human sources. Of these, 45 strains (number of strains) were isolated in Taiwan (11), UK (8), Turkey (6), Ethiopia (4), Italy (3), Hungary (3), France (2), Gambia (2), Poland (2), Lithuania (1), Spain (1), Kenya (1), and Sweden (1). The other 195 strains have been isolated in Germany from various regions. Emphasis was given on the most prevalent phage types PT8 (81) and PT4 (62). Furthermore, 16 strains belonged to phage type PT21, 9 strains to RDNC or PT6, seven strains to PT1 and six strains to PT11. Other strains (1 to 4 strains) were typed to PT1b, PT2, PT3, PT4a, PT4b, PT5a, PT6a, PT7, PT9b, PT12, PT13, PT13a, PT19, PT21c, PT26, PT32, and PT34.

Furthermore, strains isolated from two recent outbreaks caused by *Salmonella *Enteritidis were selected for outbreak investigations by MLVA [17,18]. One outbreak occurred in summer 2006 during a sporting event affecting at least 46 participants caused by contaminated pastries (éclairs). The analysis included 14 isolates from sick humans, asymptomatic carriers and contaminated pastries [17]. The other outbreak occurred in spring 2007 associated with food prepared in a kitchen of a German hospital affecting 28 patients and children of a kindergarten [18]. The analysis included 38 isolates from sick humans, asymptomatic carriers and contaminated foods.

### Sero- and phage typing

All *Salmonella *strains were serotyped according to the Kauffmann-White scheme [24] by slide agglutination with O- and H-antigen specific sera (Sifin Diagnostics, Berlin, Germany). Phage typing was performed according to Ward et al. 1987 [6].

### Selection of variable-number tandem repeats (VNTRs)

The complete genomic sequence of *S*. Enteritidis NCTC 13349 phage type 4 [25] submitted to the Tandem Repeat Finder Program Version 4.0 [26]. Repetitive regions with the highest sequence similarity of the repeats and highest number of repetitive units were selected as candidates. In addition the VNTR-locus SE-3 and SE-7 was selected from Boxrud et al. [12]. These loci contain tandem repeats with low similarity but were shown to be discriminative. The repeat motifs comprise tandem repeats from 6 to 93 bp length. The selected VNTR loci and their characteristics are shown in Table 1.

### MLVA typing

Primers were designed using the program ARRAY DESIGNER 4.0 (Premier Biosoft, Palo Alto, CA). The annealing temperature for all primers was demanded to be 55°C ± 2°C in the PCR with GC-content not less than 40%. Forward primers were labeled with FAM, HEX or TET. Sequences of primers and characteristics are shown in Table 2. The primers were multiplexed using the Qiagen PCR Multiplex Kit (Qiagen, Hilden, Germany) in a total of 12.5 μL. The PCR reaction contained 6.25 μL of the mastermix, 1.25 μL Q-solution (included in the kit), 5 pmol of the primers amplifying the loci SENTR1, SENTR2, SENTR3 and SE-7, 1.25 pmol of the primers amplifying the loci SENTR4, SENTR5, SENTR6, SENTR7 and SE-3, and 0.5 μL template cells (approx. 10^6 ^CFU/reaction). As template three to four *Salmonella *colonies grown overnight on Müller-Hinton agar (Oxoid GmbH, Wesel, Germany) at 37°C were resuspended in 200 μL double distilled water. PCR reactions were performed in a GeneAmp 9700 Cycler (Applied Biosystems). Cycling conditions were 95°C for 15 min, followed by 28 cycles of 94°C for 30 sec, 55°C for 90 sec and 72°C for 90 sec. A final extension of 72°C for 10 min was employed. A 5 μL aliquot of the PCR product was diluted 1:10 in double distilled water. A 1 μL aliquot of this dilution was mixed with 1.25 μl of the Geneflo-625-TAMRA (CHIMERx, Madison, WI) internal size marker, 0.75 μL of the 5 ng/μL concentrated TAMRA-labeled 868 bp PCR product (additional size marker) and 11 μL TS reagent (TSR) containing formamide (Applied Biosystems). The PCR product functioning as additional size marker was generated using primers paspp-flo-F and paspp-flo-B of the *floR *positive *Salmonella *strain SUO1 [27]. The samples were denatured for 2 min at 94°C and subjected to the capillary electrophoresis on an ABI-310 Genetic Analyzer (Applied Biosystems). The injection voltage was 15 kV for 15 s. The electrophoresis was run at 60°C for 45 min using POP4 polymer (Applied Biosystems) with a run voltage of 15 kV. Each peak was identified according to color and size. Each new repeat size corresponding to one locus was assigned to a distinct allele number. The allele numbers for all loci of one strain indicated the MLVA type and were entered into BioNumerics v5.1 (Applied-Maths) as character values. The following order of loci defined the MLVA type: SENTR7-SENTR5-SENTR2-SENTR6-SENTR3-SENTR4-SE3-SENTR1-SE7. The minimum spanning tree (MST) was constructed using the highest number of single locus variants (SLVs) as the priority rule. Hypothetical types were not allowed. The Nei's diversity (*D*) for each tandem repeat locus based on the 240 *S*. Enteritidis strains determined was calculated as *D *= 1 - Σ (allele frequency)^2^. Simpson's indices of diversity for MLVA and phage types were calculated according to Hunter and Gaston [28].

### Other molecular methods

Strains which were associated with the two outbreaks were ribotyped according to Liebana et al. 2001 [7] using the restriction enzymes *Pst*I-*Sph*I. Pulsed-field gel electrophoresis (PFGE) was performed according to the PulseNet protocol [29] using the restriction endonuclease *Xba*I (Roche Diagnostics, Mannheim, Germany).

Because fragment sizes determined by migration relative to standards do not always agree with the sizes predicted by direct nucleotide sequence determination, single PCR products for each locus were completely sequenced from strain 07–2642 (*S*. Enteritidis, PT4). DNA sequencing was performed by AGOWA (Berlin, Germany) using the primers shown in Table 2.

## Authors' contributions

BM conceived the study, designed the primers, supervised the experimental work, performed the analysis and wrote the manuscript. EJ carried out all experimental work. RH obtained the funding, conceived the study, helped to draft and edited the manuscript. All authors read and approved the final manuscript.
